# Focusing and sustaining the antitumor CTL effector killer response by agonist anti-CD137 mAb

**DOI:** 10.1186/2051-1426-2-S3-P95

**Published:** 2014-11-06

**Authors:** Elixabet Bolaños-Mateo, Bettina Weigelin, Alvaro Teijeira, Ivan Martinez-Forero, Sara Labiano, Arantza Azpilicueta, Aizea Morales-Kastresana, Jose I Quetglas, Esther Wagena, Alfonso Rodriguez, Lieping Chen, Peter Friedl, Ignacio Melero

**Affiliations:** 1 Center for Applied Medical Research, Pamplona, Spain; 2 Radboud University Nijmegen, Nijmegen, Netherlands; 3 CIMA, University of Navarra, Pamplona, Spain; 4 NIH - National Cancer Institute, Bethesda, MD, USA; 5 Yale University, New Haven, CT, USA

## 

B16-derived OVA-expressing melanomas resist curative immunotherapy with either adoptive transfer of activated anti-OVA OT1 cytotoxic T lymphocytes (CTLs) or agonist anti-CD137 (4-1BB) mAb. However when acting in synergistic combination, these treatments consistently achieve tumor eradication. Tumor-infiltrating lymphocytes that accomplish tumor rejection exhibit enhanced effector function in both transferred OT-1 and endogenous CTLs. This is consistent with higher levels of expression of eomesodermin in CTLs and with confocal microscopy evidence for more efficacious tumor-cell killing. Combined immunotherapy of tumors monitored by intravital live-cell two-photon microscopy reveals persistence of the OT1 CTL-effector phenotype over prolonged periods of time. Anti-CD137 mAb delayed loss of function with focused and confined interaction kinetics of OT-1 CTL with target cells lasting up to ten days post-transfer. The synergy of adoptive T cell therapy and anti-CD137 mAb thus results from in-vivo enhancement of effector functions.

